# Arranging university semester date to minimize annual CO_2_ emission: A UK university case study

**DOI:** 10.1016/j.isci.2021.103414

**Published:** 2021-12-08

**Authors:** Zihao Li, Wei Sun, Yue Xiang, Camilla Thomson, Gareth Harrison

**Affiliations:** 1 TUEIRI Tsinghua University Energy Internet Research Institute, Chengdu, China; 2 School of Engineering, University of Edinburgh, Edinburgh, UK; 3 College of Electrical Engineering, Sichuan University, Chengdu, China

**Keywords:** Energy resources, Energy policy, Energy flexibility, Energy systems

## Abstract

Existing methods of reducing carbon emissions on campus often require substantial investment, and the potential opportunities for carbon dioxide and energy savings in universities with existing infrastructure have not been considered in much detail. This work fills this gap by considering an indirect and soft demand response strategy, i.e., semester arrangement. To identify the optimal operational strategy of a realistic campus-level multi vector energy system (MES) in Scotland based on CO_2_ emissions, an original tool is presented. Two conclusions can be drawn safely from the case study. Firstly, changing the operational mode of the university could significantly reduce CO_2_ emissions. Secondly, considering the difference between average emission factor (AEF) and marginal emission factor (MEF) in the power grid, the different operational modes will bring different electricity/heat demands and also affect carbon emissions. The work opens up a new perspective for worldwide university operators who are considering reducing CO_2_ emissions.

## Highlights

• An energy hub model to minimize CO_2_ emission for a real university is formulated
• The impact of semester schedules on the annual campus-level CO_2_ emissions is examined
• Thousands of potential arrangements are compared to identify the eco-friendly mode
• The effects of dynamic carbon emission factors on CO_2_ calculations are being discussed

## Graphical abstract

**Figure:**
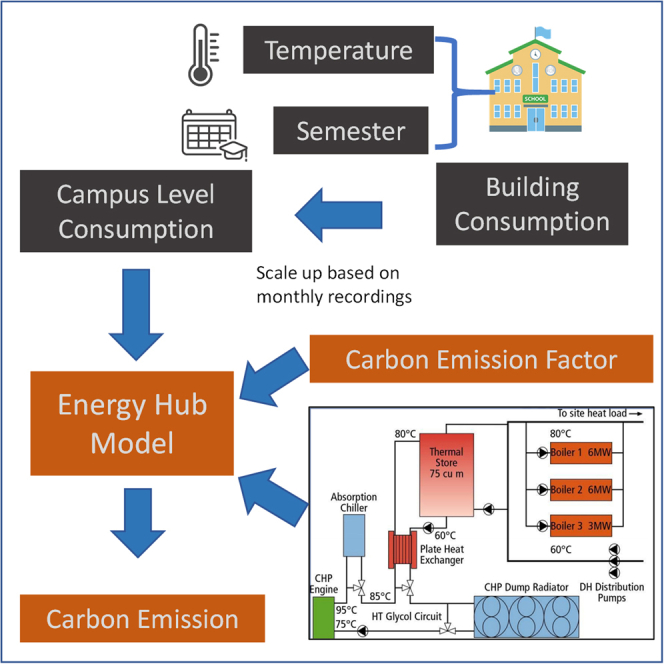

## Introduction

Facing the challenge of global warming, governments worldwide have introduced relevant policies to reduce greenhouse gas emissions. For instance, China plans to achieve carbon neutral by 2060 (Harvey, 2020). The UK government has announced to reduce the overall carbon emissions to net zero by 2050 (Walker et al., 2019).

Besides the governments, large organizations across various sectors of society are also working hard to reduce their own carbon emissions. In education sector, as representatives for advanced technology and scientific research, a large number of universities have set their own goals in terms of energy conservation and CO_2_ emissions. Over 700 universities in the USA have joined the American College and University Presidents' Climate Commitment, an initiative that aims to reduce carbon emissions to achieve carbon neutral (Dyer and Dyer, 2017). Similar low carbon targets have also been published by a number of British universities: most of them have promised to reduce their emissions by 80% by 2050 on the 1990 CO_2_ emission baseline (EAUC, 2019). However, several top-tier universities have demonstrated a further ambitious but concrete goal, achieving either the “net zero emission campus” or “carbon neutral campus,” around the middle of this century. Table 1 lists some universities that publish zero-carbon targets (EAUC, 2019).

**Table 1:** Universities within QS ranking 100^th^ that have proposed zero-carbon targets

QS 100 institutions	Carbon reduction target
The University of Nottingham	Net-zero by 2028
Cardiff University	Carbon neutral (scope 1 and 2) by 2023
Newcastle University	Net-zero carbon by 2040
University of Leeds	2030 Net-zero carbon
University of Glasgow	Nearly zero carbon 2050
University of Warwick	Net-zero carbon by 2050
University of Bristol	Carbon neutral by 2030
London School of Economics	Net-zero greenhouse gas emissions 2050
King's College London	Carbon neutral by 2025
The University of Manchester	Zero carbon by 2038
The University of Edinburgh	Net-zero carbon by 2040
University College London	Carbon neutral (all scopes) by 2030
University of Cambridge	Carbon (scope 1 and 2) to zero by 2048

With universities putting forward their ambitious plans, researchers have made great efforts on establishing many energy optimization models aimed at reducing greenhouse gas emissions in campuses. For example, researchers in Cornell University addressed a new geothermal-biomass system, which will cut CO_2_ emissions by 94,000 metric tons (43% existing emission of the campus) with millions of dollars (Beckers et al., 2015). Nevertheless, the specific measures mentioned in these papers, such as demand reduction through targeted upgrades to buildings and installation of new generation or conversion, often require a large amount of investment and an accurate estimation to hourly loads of universities. This is a considerable challenge for the management and financial strength of most British universities. Consequently, there is value in looking at noninvestment measures to help universities to reduce CO_2_ emissions, for instance, changing the operation of campus systems to use the power from the utility grid when the grid CO_2_ intensity is at a relatively low level or modulating consumption through demand-side management offer scope. In fact, the increasingly clean power grid has already helped colleges and universities to save energy and reduce green gas emissions. But existing studies have not dealt with how to take advantage of it in the medium to long term (Times Higher Education, 2019).

At present, universities around the world, especially in developing countries, do not have a clear roadmap of how to reduce their own carbon emissions. Activities in colleges and universities often heavily rely on interpersonal communication, and their behaviors are regulated by teaching tasks and holidays, making it difficult to carry out traditional load-side management, e.g., shitting demands by hours to reduce carbon emissions.

This paper, using real historical data, investigates a campus-level multi vector energy system (MES) model, which examines dynamic CO_2_ intensity and semester schedule influences on the annual campus-level CO_2_ emissions. Due to data limitation, i.e., no daily energy consumption on the campus level, an on-campus building demand is scaled up according to monthly recordings and used as input to the MES model. The details of the building demand model are described in the STAR method, which successfully integrates the semester arrangement as one of the model inputs. Compared with previous MES studies that minimize CO_2_ emissions from the energy system perspective, this paper conducts a novel applied study in large educational organizations, i.e., UK universities, and contributes novel research insights into what extent does the make-up of the academic year affect the university's annual carbon emissions. The proposed method can also be applied to other universities in the UK and also worldwide. In the case study, more than 1,800 potential university semester arrangements were compared to identify the most environmentally friendly mode for the University of Edinburgh (UoE). The contributions mainly are as follows:

A) Establishing an MES model with the goal of minimizing carbon dioxide emissions for a real university campus.
B) Providing an insight into how CO_2_ emissions differ when different emissions factors are applied, specifically annual fixed average emission factor (AEF), weekly average emission factor (AEF), and weekly marginal emission factor (MEF), which does not appear in the existing research.

### Carbon emission factors

In the past decade, Great Britain has made tremendous progress in decarbonizing its power grid. With the high penetration of renewable generation, grid carbon average emission factor (AEF) has decreased from over 400 gCO2/kWh to 250 gCO2/kWh around and is expected to further decrease to well below 100 gCO2/kWh by 2030 (Deben et al., 2015). AEF refers to an index obtained by weighting the average carbon content of different generating sets in each time period. The lower the index, the higher the proportion of nuclear and renewable energy on the power generation side. However, for end-users, this index cannot fully favor the efforts in energy conservation and emission reduction. Researchers (Hawkes, 2010; Thomson et al., 2017) point out that changes in demand does not affect all the generators in the power system equally but tends to affect specific generators. The metric that estimates changes in grid CO2 intensity due to demand variation is called the marginal emissions factor (MEF), and it is a function of specific CO2 intensity of the set of generators that respond to that intervention. In economics, it is often viewed as the marginal generating unit (the cheapest power plant that still has spare capacity), but in practice, it is complicated by the need to maintain secure operation. Compared with AEF, MEF is widely defined as the incremental change in carbon emissions as a result of a change in demand (Hawkes, 2010; Thomson et al., 2017). This is a useful index for analyzing fuel cost benefits and emission benefits from changes in demand patterns.

Due to different power generation combinations, AEF and MEF may show seasonal differences. For example, solar photovoltaic (PV) generates more electricity in the summer than winter, which may result in lower AEF and MEF. Besides, different local power networks have distinct levels of carbon intensity. The AEF of Scotland's electricity grid, the cleanest in the UK, has fallen from 318 gCO2/kWh in 2010 to 54 gCO2/kWh by 2016 (Scottish Government, 2018), whereas the decarbonization in England is slower but the AEF may also drop below 50 gCO2/kWh as well by 2050 (Deben et al., 2015). Overall, as power system physical quantities, both MEF and AEF are greatly driven by the generation combinations that vary with time and space, which creates a great number of uncertainties to calculate in reality.23

### University operational mode

Since the power grid carbon intensity changes with time but the natural gas CO_2_ intensity is a relatively fixed value, we can establish an “operational mode-demand-CO_2_ emission model” for universities. The model can help the university to find a mode of operation with minimal CO_2_ emissions. To achieve this, it is required to review the operational mode of universities in the UK.

The exact start and end date of the academic year and vacation times vary among UK universities. Table 2 lists UK universities with QS rank under 100^th^ and their semester arrangements for 2019/2020. It is clear that different universities prefer different arrangements, which suggest thousands of permutations in theory. Also, some key points that are useful in the following modeling can be found in Table 2. Firstly, there are significant similarities between universities’ holiday arrangements. The December/January break often lasts for 3 to 4 weeks, which always involves two important festivals, i.e., Christmas and New Year Eve. Meanwhile, summer vacation normally begins from late May or June until the middle of September. As a result, the learning weeks usually take up to 30 weeks in almost all UK universities. However, the distribution of learning weeks differs between the English and Scottish systems. More specifically, universities in Scotland tend to divide learning weeks equally in winter and spring, leaving 3 to 4 weeks for exams in summer, e.g., Universities of Edinburgh and Glasgow (The University of Edinburgh, 2020; University Of Glasgow, 2020). Others prefer to arrange the study time evenly with summer semester tending to be extended to 8 or 9 weeks, e.g., Imperial College and University College London (Imperial College London, 2020; University College London, 2020). Finally, the start date of the semester in every university is also different between the start of September to the second week of October. Based on the above information, we can get a good indicator of all the potential alternative semester arrangement combinations of UK universities.

**Table 2:** Semester arrangements in some UK universities

University name	Autumn term	Spring term	Summer term
University of St Andrews	9/09–21/12	27/01–16/03	30/03–1/06
University of Southampton	30/09–14/12	6/01–13/03	20/04–13/06
University of Leeds	30/09–13/12	13/01–27/03	27/04–19/06
University of Birmingham	30/09–13/12	13/01–27/03	27/04–19/06
The University of Sheffield	23/09–20/12	20/01–5/04	27/04–13/06
Durham University	20/09–13/12	13/01–20/03	27/04–26/06
University of Glasgow	16/09–20/12	13/01–27/03	27/04–29/05
University of Warwick	23/09–7/12	6/01–14/03	20/04–27/06
University of Bristol	30/09–20/12	13/01–30/03	30/04–26/06
London School of Economics	30/09–13/12	13/01–3/04	4/05–19/06
King's College London	24/09–13/12	7/01–29/03	22/04–31/05
The University of Manchester	26/09–13/12	13/01–27/03	19/05–9/06
The University of Edinburgh	09/09–20/12	13/01–3/04	27/04–22/05
University College London	28/09–13/12	13/01–3/04	35/04–26/06
Imperial College London	23/09–13/12	13/01–27/03	27/04–12/06
University of Cambridge	08/10–6/12	14/01–13/03	21/04–12/06
University of Oxford	14/10–7/12	20/01–14/03	26/04–20/06

University Of St Andrews, 2020; University Of Southampton, 2020; University Of Leeds, 2020; University of Birmingham, 2020; The University Of Sheffield, 2020; Durham University, 2020; University Of Glasgow, 2020; University Of Warwick, 2020; University Of Bristol, 2020; London School of Economics and Political Science, 2020; King's College London, 2020; Manchester University, 2020; The University of Edinburgh, 2020; Imperial College London, 2020; University of Cambridge, 2020; University of Oxford, 2020.

From the probable routes, considering the start date (four Mondays) and semester arrangement difference, there are currently over 1,800 possible university semester arrangements. A specific semester arrangement is defined by not only the number and the order of learning weeks and holidays but also by the date on which it begins. For example, for two identical schedules, they will be regarded as two different arrangements if one starts on September 8^th^ and the other starts on October 8^th^. Our previous work shows that according to the time in the semester, people with different identities behave differently and affect the load of the buildings (Li et al., 2020).

Overall, semester arrangement, or the university operational mode, can be regarded as a kind of collective users' behavior, which will influence the energy consumption. By taking a set of possible university operational modes as inputs, a group of energy consumptions generated by different operational modes can be obtained. Then, the corresponding CO_2_ emission is calculated through the corresponding dispatch model that describes the energy system on campus. Finally, based on the results, the most “eco-friendly” operational mode for the university could be identified.

### Balancing demand: UK university energy systems

Some world-famous universities have proposed their own strategy and actions on how to minimize the CO2 emission (White, 2014). The universities in the UK are not exceptional. In fact, many UK universities have already established a basic low carbon energy system to meet all or part of their cooling, heating, or electricity demand. Table 3 below shows the existing onsite energy facilities in some UK universities. It is obvious that CHP and Solar PV are common but whether they can fully rely on these existing facilities to achieve the 2050 target is a key question.

**Table 3:** Existing onsite energy equipment for selected UK universities

University name	Existing energy facilities
University of St Andrews	Solar PV; ground source heat pump; CHP
University of Southampton	CHP； gas boiler
University of Leeds	NA
University of Birmingham	CHP
The University of Sheffield	CHP
Durham University	CHP; buy electricity from wind farm
University of Glasgow	CHP
University of Warwick	CHP; solar PV
University of Bristol	CHP; solar PV
London School of Economics	CHP; solar PV
King's College London	CHP； solar PV; buy electricity from wind farm
The University of Manchester	CHP; solar PV; gas boiler
The University of Edinburgh	CHP; solar PV; gas boiler; absorption chiller
University College London	CHP
Imperial College London	CHP； gas boiler
University of Cambridge	CHP； solar PV; buy electricity from wind farm
University of Oxford	CHP; solar PV; gas boiler

University Of St Andrews, 2020; University Of Southampton, 2020; University Of Leeds, 2020; University of Birmingham, 2020; The University Of Sheffield, 2020; Durham University, 2020; University Of Glasgow, 2020; University Of Warwick, 2020; University Of Bristol, 2020; London School of Economics and Political Science, 2020; King's College London, 2020; Manchester University, 2020; The University of Edinburgh, 2020; Imperial College London, 2020; University of Cambridge, 2020; University of Oxford, 2020.

For these established campus energy systems, without considering massive equipment investment, energy saving and emission reduction are achievable by adjusting the electricity and heat demand through demand response. However, such actions require a very clear and deep understanding of the university's demand structure. The load and the activities behind it have to be categorized so that the universities can recognize what is a potential flexible load. Unfortunately, there is few convincing relevant researches to give an answer. In fact, the suggestion of changing semester schedules is actually a soft form of demand response approach that requires neither additional investment nor understanding of university demand structure. Thus, it can be easily implemented in almost all UK universities to impact their load and carbon emissions.

### Low carbon energy hub review

There has been significant work in literature on integrating different energy resources into one platform in energy system research. In recent years, the concept of energy hub presented in the literature (Geidl et al., 2006) has been widely applied in the comprehensive analysis of multi-vector energy systems worldwide. An energy hub is taken as a unit where multiple energy carriers can be converted; a typical energy hub consumes electricity and natural gas at its input side and provides the electricity, heating, and cooling energy services at output side (Mancarella, 2014).

Extensive researches are carried out on the energy hub, especially toward low carbon target. Hurwitz et al. (Hurwitz et al., 2020) develop a building level MES operational model that uses linear approximations to successfully describe nonlinearities in the efficiencies of energy conversion processes. The case study is optimized to reduce costs based on representative seasons and carbon tax scenarios for a campus in the USA. Similarly, scientists from University of Vermont's (Almassalkhi and Towle, 2016) present piece-wise linear modeling to capture nonlinear converter efficiencies, the case study that uses University of Vermont's campus to demonstrate that the traditional hub models can significantly undersize energy storage, as compared with the more accurate piece-wise linear energy hub formulation. In addition, Olsen et al. (Olsen et al., 2018) extends the analysis of low-carbon energy hub design with two strategic scenarios: the first scenario makes investment decisions while accounting for a hub operator that may ignore emissions-reduction goals; the second scenario determines carbon prices to induce lower-emission investment and operating decisions. The energy hub model concept has been widely adopted worldwide to analyze various applied research questions in the context of energy system integration. Apart from the USA, Europe researchers investigate models to generate low carbon future scenarios. They address a novel and powerful model that can simultaneously determine the design and operation of integrated multi-vector energy networks comprising technologies for conversion, storage, and transport. The model is used to consider a number of scenarios for Great Britain low carbon future in 2050 (Samsatli and Samsatli, 2018). Besides, Chinese scientists (Cheng et al., 2018) propose a bilevel expansion planning model of MES that considers the emission constraints under a decentralized approach. The upper-level model investigates the optimal planning scheme for integrated power and natural gas networks in the multi-regional MES.

Although there are many papers that investigate energy hub model to reduce carbon emissions, most of them archive the reduction either by selecting and sizing the optimal conversion equipment or by dispatching the optimal use of these pieces of equipment; few has considered how to reduce carbon emission by changing the schedules of some large organizations that have significant changes in energy usage between periods over a year, for example, university and schools. This paper aims to fill the gap by integrating semester arrangement into an energy hub model to investigate university activities impact on carbon emissions. Apart from the semester arrangement, AEF and MEF effects on CO_2_ calculation are also discussed, which do not appear in most acknowledged publications.

### From energy consumption to CO_2_ emission

According to the demand data provided by the “operational-demand model,” the corresponding CO2 emissions can be calculated by different methods (Thomson et al., 2017). The choice of CO2 emission factor will have a major impact on the value and reliability of resulting CO2 emissions. For example, 1 kWh electricity from the utility grid is associated with disparate amounts of CO2 in summer and in winter because of different seasonal generator combinations. Were summer demands to shift to winter, the change in terms of carbon emissions would not be linear with the change in energy consumption. Consequently, CO2 emission factor is crucial in demand-CO2 emissions calculation. Nevertheless, existing universities do not use the actual CO2 emission factor when they calculate their CO2 emissions, as emissions reporting regulations mandate the use of official annual AEF values; this could overestimate or underestimate the true CO_2_ emissions. This paper, in the case study, will show how CO_2_ emissions vary when different emissions factors are implemented.

## Results

### Methodology framework

**Figure 1:**
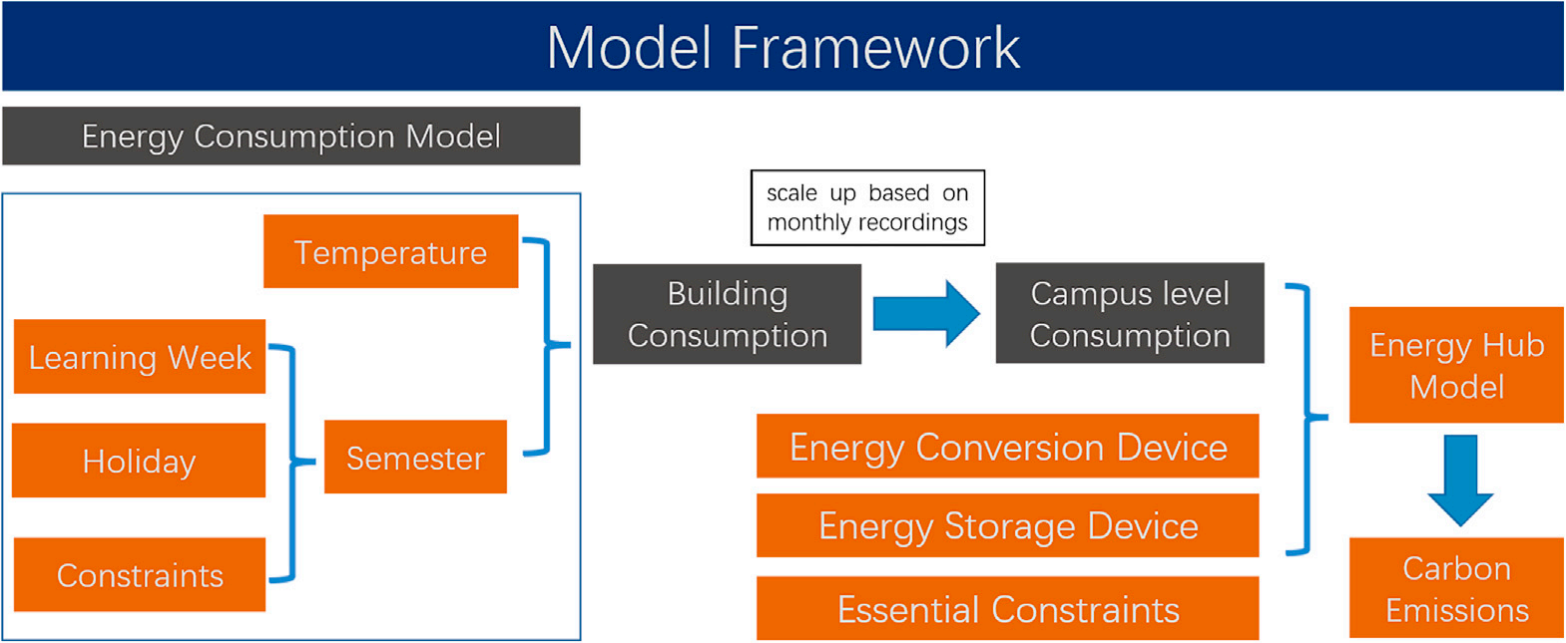
Model framework

This work is conducted on a series of sub-models. Figure 1 illustrates how the module of energy hubs, CO_2_, building loads, and semester dates/constraints are included in the modeling of the problem. The first step is to generate semester arrangements from a series of constraints (details in the STAR method section), then together with temperature, being used as inputs to the building model. The building model will give building level daily energy consumption (both heat and electricity) and scale up based on monthly campus energy meter recordings to get campus-level energy consumption, which is used as input to the energy hub model. The energy hub model, with the objective of minimum carbon emissions, will determine the least carbon emissions that can be achieved for different semester scenarios. The recommendation of semester arrangements is finally made by comparing their carbon emissions found in the previous step.

### Demand data obtained

For the purpose of finding the optimal CO_2_ saving operational mode, the electricity and heat/cooling results from our previous work are modified and used as a demand time series for the campus energy system. A full description of the model is found in the STAR Methods section.

Due to data availability, the original model is designed to describe energy consumptions in a mixed-use building of UoE, the Chrystal MacMillan Building (CMB), rather than for the whole campus. Here, the campus-level demand is obtained by enlarging the results from that building. The scaled-up results keep the annual consumption the same as the real recordings. It may not be able to precisely reflect daily campus-level consumption patterns. Nevertheless, the curve obtained should be good enough to analyze the semester arrangement’s impact on campus energy consumption.

Because the total annual energy consumption results in this study come from an identical model, i.e., LMSR (Linear model stepwise regression) model from an existing paper (Li et al., 2020), under the condition of the same carbon emission factor, the daily emission of CO_2_ fully depends on the daily load size, which is driven by the semester arrangement. That is to say, the CO_2_ emission difference between different operational modes is purely from semester arrangement differences and has nothing to do with other factors, such as model accuracy. As a result, the results can be used as a benchmark to determine the extent that semester arrangements can help universities to save energy and reduce emissions.

### Energy hub model

The demand model can generate different load distributions based on different semester information but cannot get a corresponding supply side generation combination. Thus, an energy system model is necessary to identify different generation combinations that can match the load, which is also the basis of the CO_2_ emission calculation.

**Figure 2:**
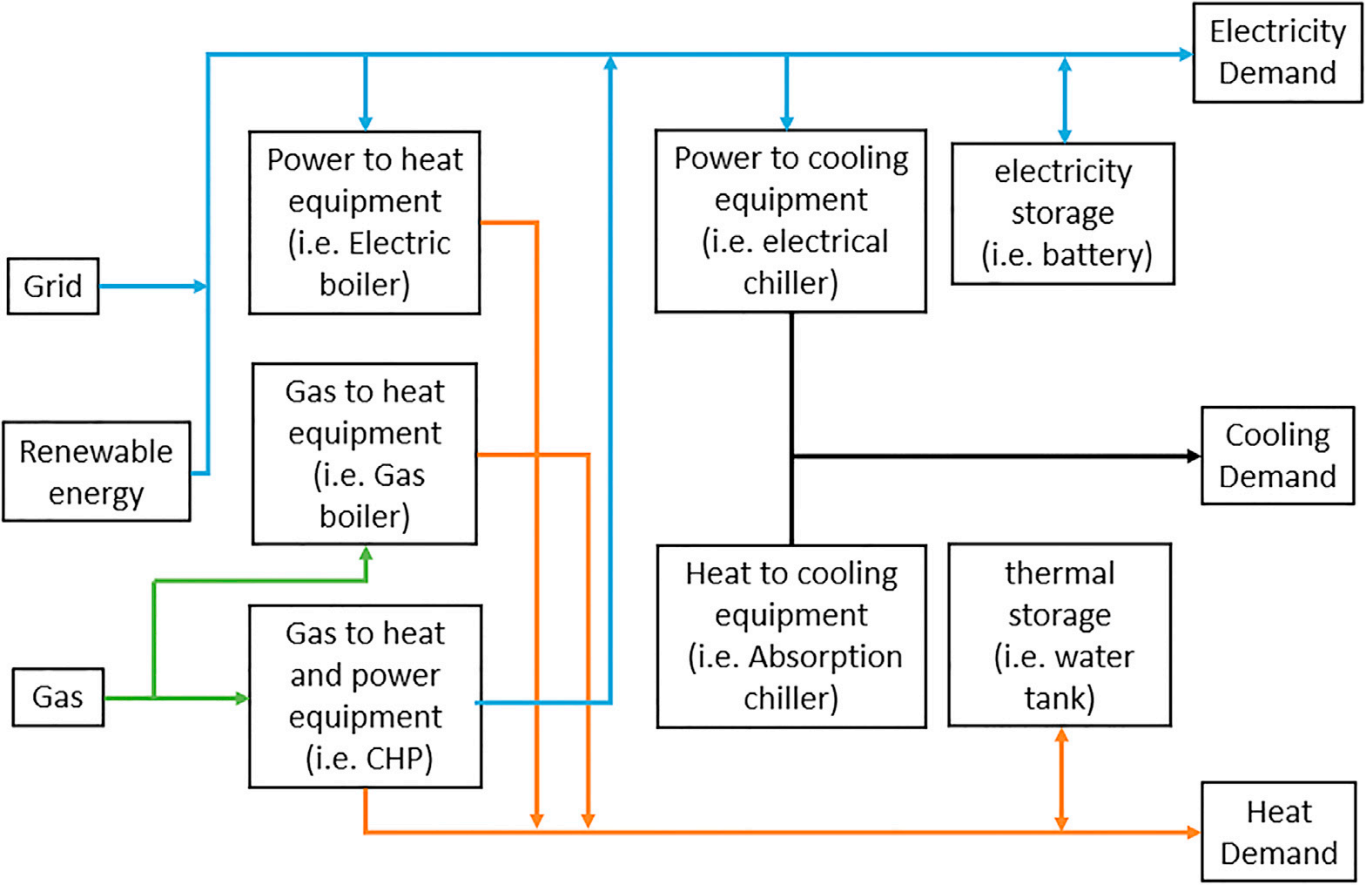
Example of a simplified overview of campus energy hub model

For different campuses in the UK, there may be different combinations of equipment. However, as shown in Figure 2, a campus energy hub model normally contains three parts: (1) energy supply, such as natural gas, and electricity from the utility grid; (2) energy conversion technology, including combined heat and power plant (CHP), gas boiler (GB), absorption chiller (AC), etc.; and (3) energy storage, including electricity storage (ES), heat storage (HS), and cooling storage (CS), although the last one normally does not appear in UK university campuses because the average UK cooling demand is relatively small. The mathematical formulation of the proposed energy hub model for university campuses is detailed in the STAR method section.

### Case description

In the UK, there are many universities located in different regions with different energy consumption curves and energy conversion technologies. This case study uses the George Square campus (GS) in UoE as an example, by comparing CO_2_ emissions in different operational modes. The energy hub model for GS campus is coded in MATLAB 2018a (Matlab, 2018) with YALMIP (Lofberg, 2004) and solved by CPLEX (IBM, 2015). The specific model for the GS campus may not work directly for other campuses in the UK, but such a method is generally applicable.

**Figure 3:**
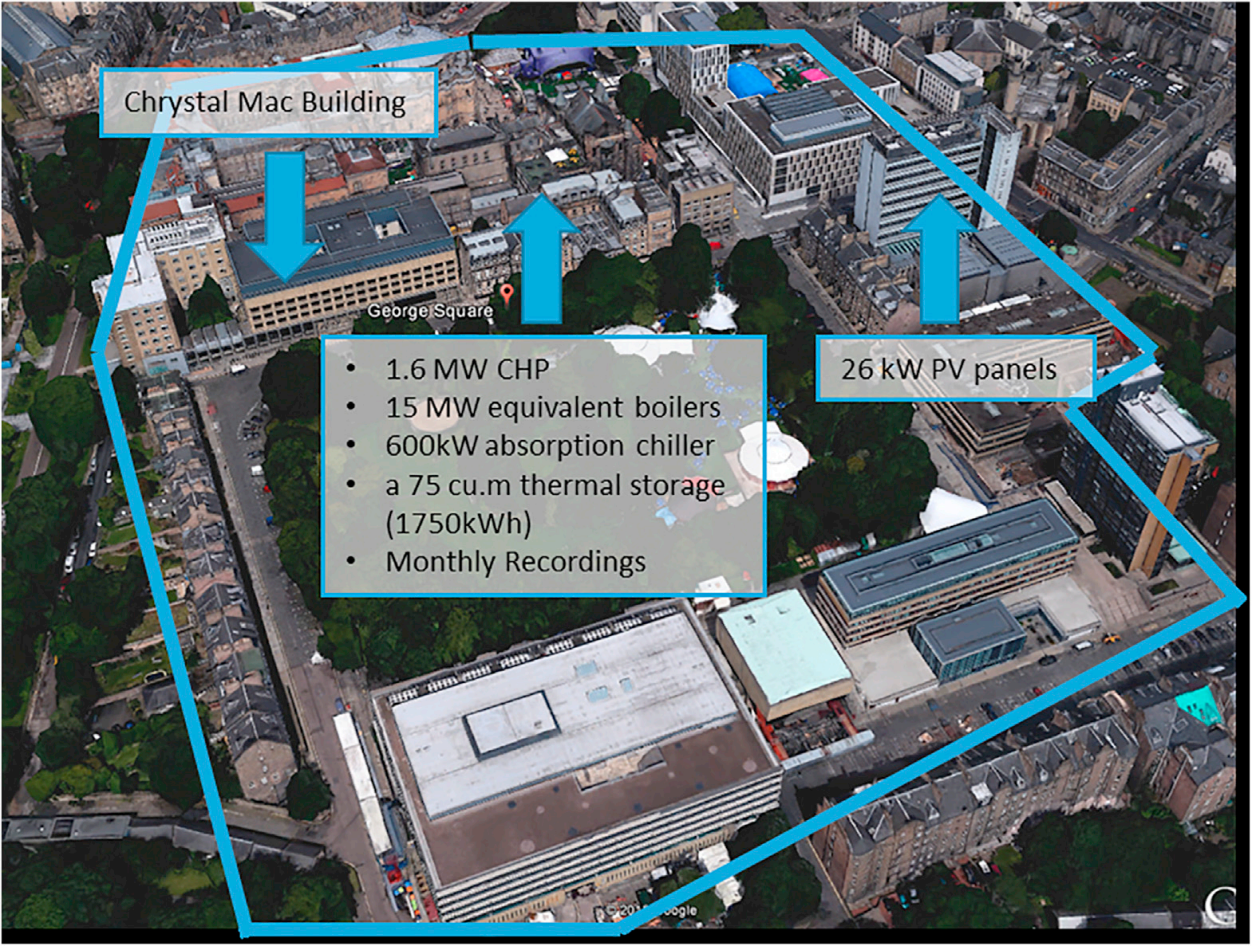
GS campus overview

The GS campus, the most important center of the university with over 10,000 students, is located in the heart of Edinburgh city. GS has 21 buildings linking to a 1.6 MW CHP station, a 26 kW PV system, a 15 MW GB, and a 1750 kWh HS. Meanwhile, the campus has a 600 kW AC for summer cooling in some buildings and laboratories (University of Edinburgh, 2006). Figure 3 shows the global view of that campus.

This paper created daily campus energy consumption by scaling up the modeled CMB daily energy consumption. The GS campus’s typical annual total power and gas use is 17,000 MWh and 20,000 MWh, respectively.

**Figure 4:**
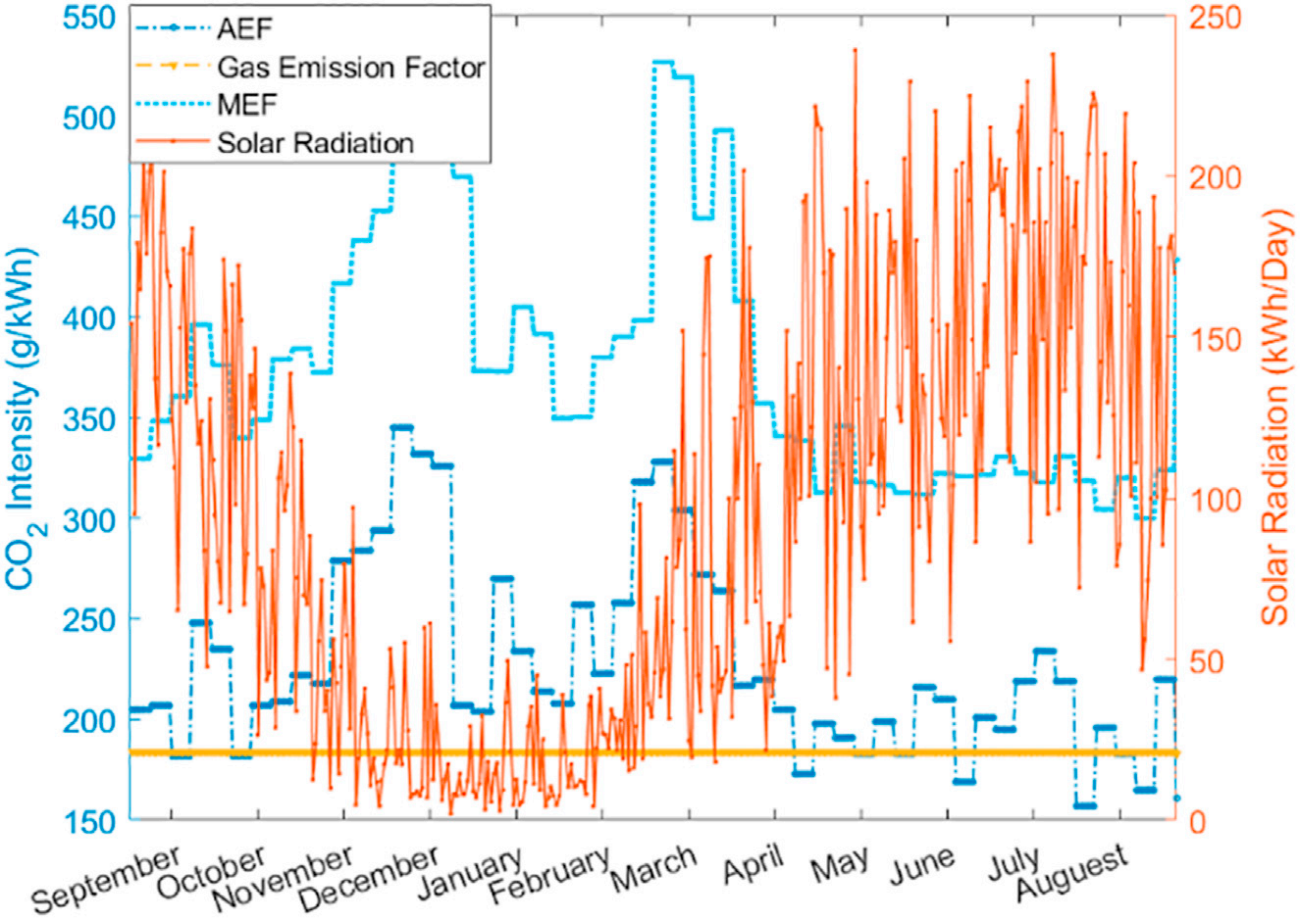
Carbon intensity and solar radiation data

Table 4 lists the technical parameters, which are given by the university operator, of the energy conversion facilities at GS campus: CHP, GB, AC, HS, PV. It is emphasized that due to the data limitations, the Scottish grid monthly AEF and MEF are not available. As a consequence, this study uses 2015 September to 2016 September UK AEF and MEF data from Electric Insight, which is based on data from National Grid and Elexon (Figure 4) (Staffell et al., 2017). By demonstrating the entire annual demand curve on the website, the weekly AEF and MEF are automatically shown up. To be more specific, AEF and MEF are acquired by reading the value from “*Environment-Emission*” and “*Dispatchable and Flexible*” on the website, respectively. Using UK level datasets not only avoids the complication of estimating local AEF or MEF but also brings another advantage: for other UK universities that may use this approach in the future, national-level data will lead to comparable results. Otherwise, it is difficult to compare different university operational modes’ influence on CO_2_ emissions because of local power network differences. Figure 4 also shows the solar data (Pfenninger and Staffell, 2016). Table 5 shows the range of historical semester arrangements in UoE and other UK universities.

**Table 4:** Equipment parameters

Technology	AC	GB	CHP	PV	HS
Output type	Cooling	Heat	Electricity/Heat	Electricity	Heat
Input type 1	Heat	Gas	Gas	Solar	Heat
Efficiency to output type 1/type 2	0.7	0.89	0.4/0.45	0.18	0.96
State of charge per day	0–1	0–1	0–1	0–1	0–1
Capacity (kW)	600	15,000	1,600	26	1,750

**Table 5:** Existing UK universities arrangements in history

Semester arrangement	Other UK university semester length	UoE semester length
Semester start time	Sept. 8–Oct.8	Third week of Sept.
Autumn & winter learning time	10–14 weeks	14 weeks
Winter vacation	2–4 weeks	2 weeks
University closure time	1–2 weeks	2 weeks
Spring learning time	8–13 weeks	12 weeks
Spring vacation	2–4 weeks	2 weeks
Summer learning time	3–9 weeks	4 weeks
Summer vacation	10–16 weeks	16 weeks
Total study week	28–30 weeks	30 weeks
Total week	52 weeks	52 weeks

University Of St Andrews, 2020; University Of Southampton, 2020; University Of Leeds, 2020; University of Birmingham, 2020; The University Of Sheffield, 2020; Durham University, 2020; University Of Glasgow, 2020; University Of Warwick, 2020; University Of Bristol, 2020; London School of Economics and Political Science, 2020; King's College London, 2020; Manchester University, 2020; The University of Edinburgh, 2020; Imperial College London, 2020; University of Cambridge, 2020; University of Oxford, 2020.

See Table S1 for the full list of potential semester arrangements in details.

### Scaled up LMSR demand modeling results

**Figure 5:**
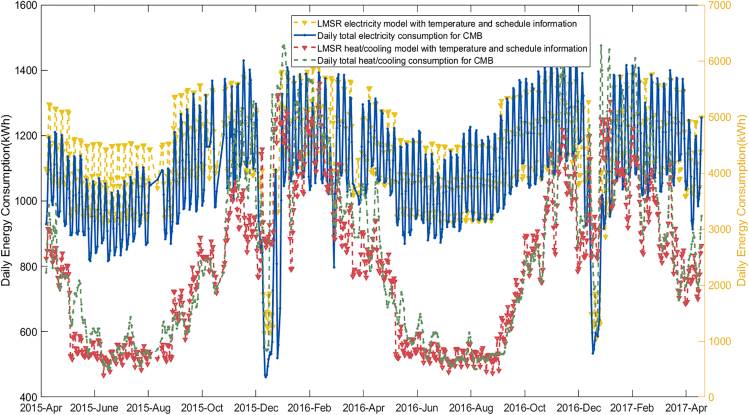
LMSR electricity heat/cooling demand modeling results

**Figure 6:**
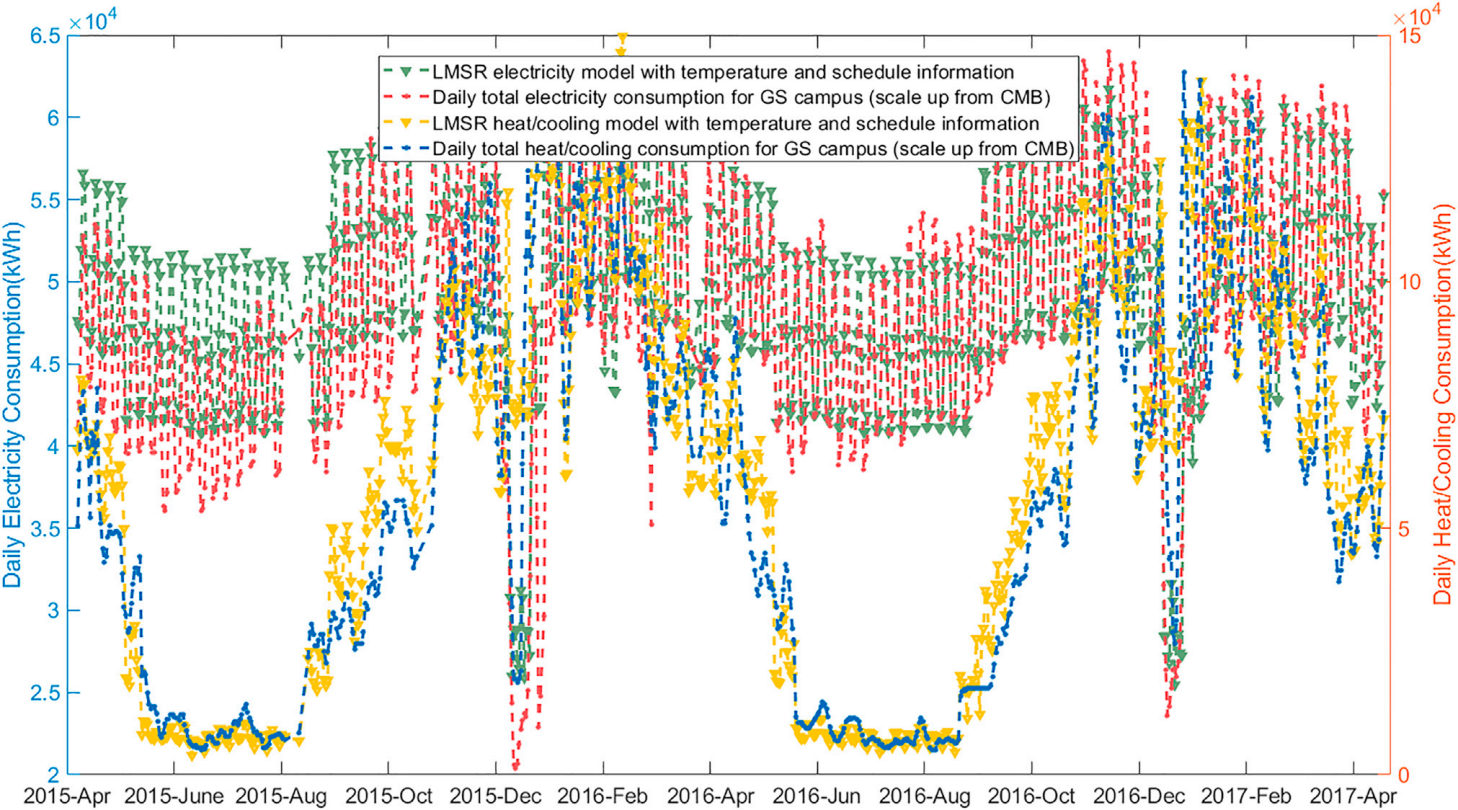
University scale daily energy consumption

The LMSR model is to predict heat load and power load according to different semester arrangements, provided a basis for the energy hub model (Li et al., 2020). The model is designed for a mixed-use building, CMB, and it clearly captures its daily energy consumption patterns in Figure 5, and this is shown scaled up to campus level in Figure 6.

In the UK, besides the UoE existing operational mode, there are over 1800 different other potential combinations of weekly operating modes (Tables 5 and S1). By assuming the outdoor temperature remains unchanged, all potential modes are used as inputs to the LMSR model to corresponding energy consumption results.

**Figure 7:**
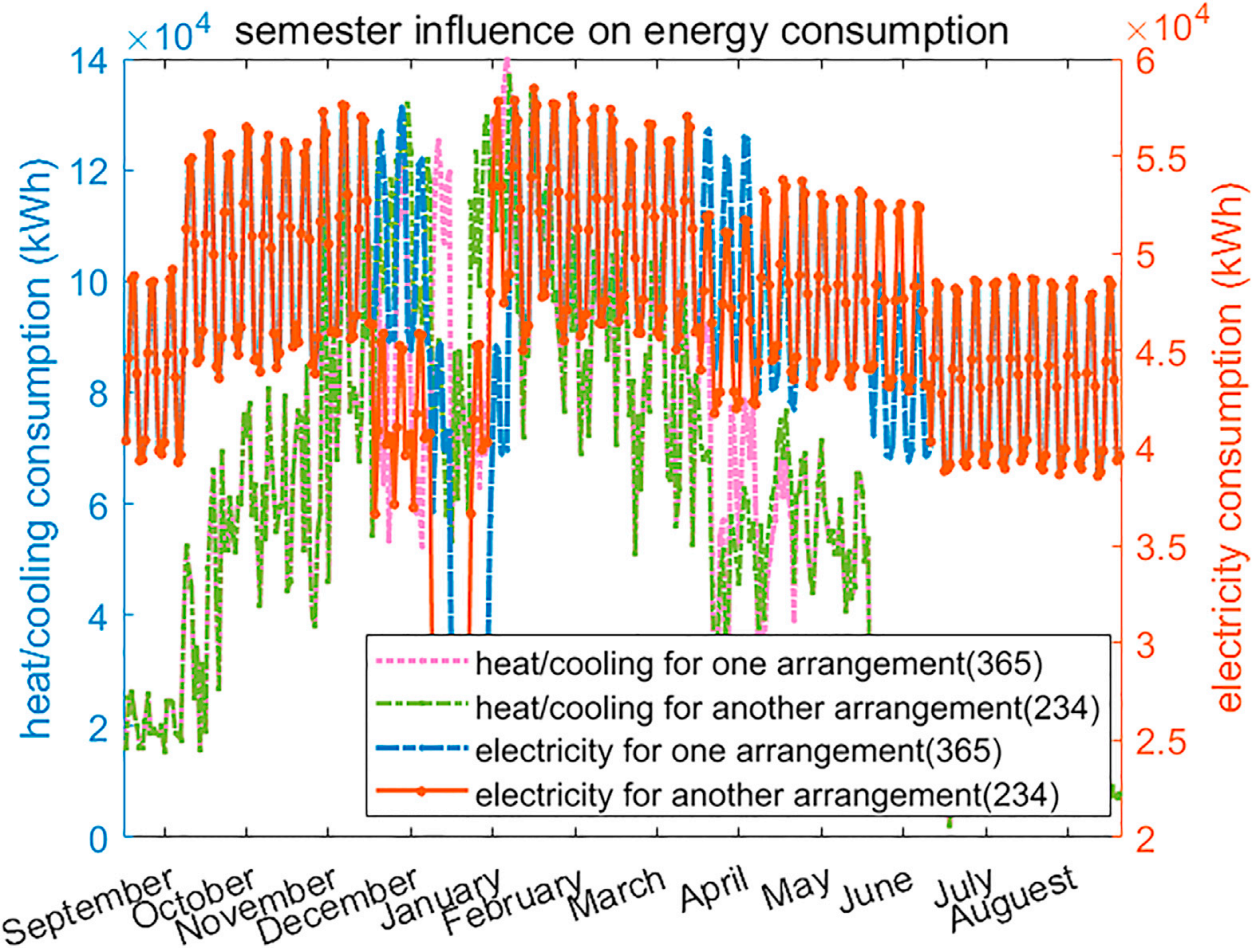
Energy patterns in different arrangements

**Figure 8:**
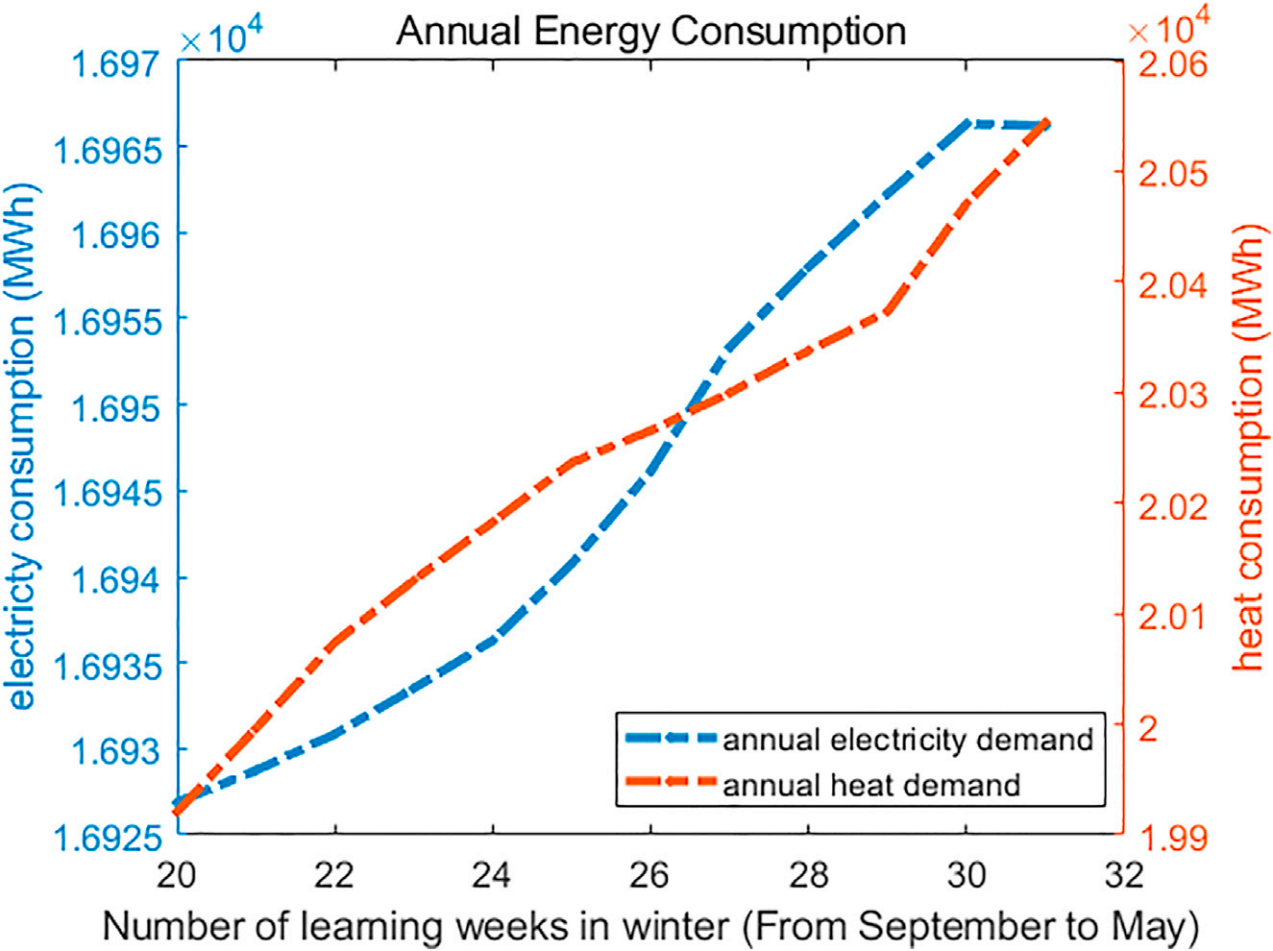
Energy consumption relationship with winter semesters

After comparing the obtained energy consumption, two conclusions can be drawn safely. Firstly, different semester structures will bring different demand patterns. Figure 7 illustrates what happens to the electricity and heat consumption profiles when semester arrangement changes (#234 and #365 are different semester arrangements, which can be checked in the Table S1). Secondly, Figure 8 shows that as more teaching tasks are scheduled in the winter period (defined as September to May), heat and electricity demands increase. Therefore, to minimize energy demand, it would be necessary to arrange teaching activities as smoothly as possible throughout the year, i.e., reducing winter courses and adding more summer courses to the existing operational mode.

### Energy hub results and AEF/MEF analysis

#### Semester schedule relationship with CO_2_ emissions

**Figure 9:**
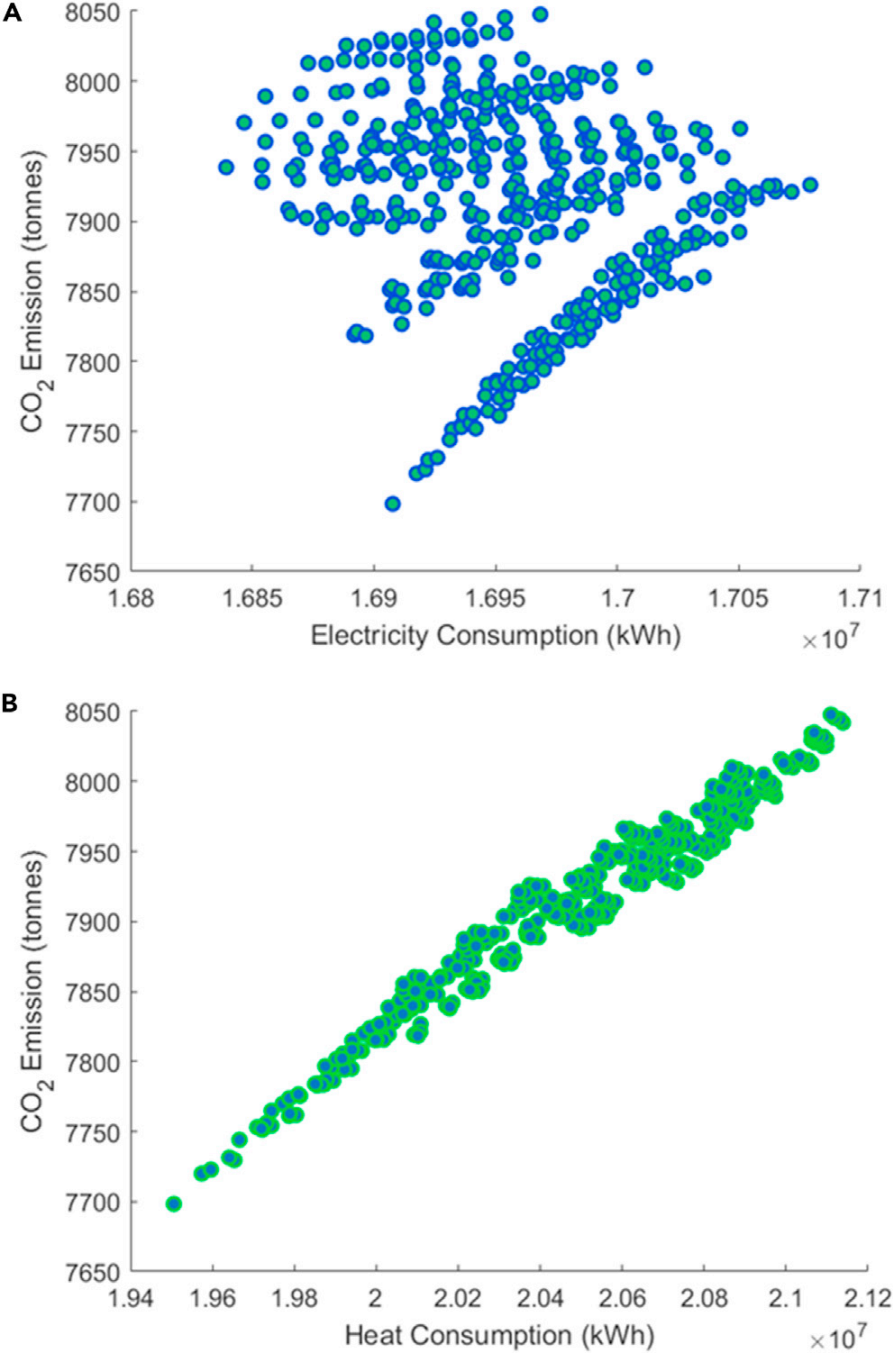
Energy consumption with CO2 emission (AEF) (A and B) (A) Electricity and (B) heat.

**Figure 10:**
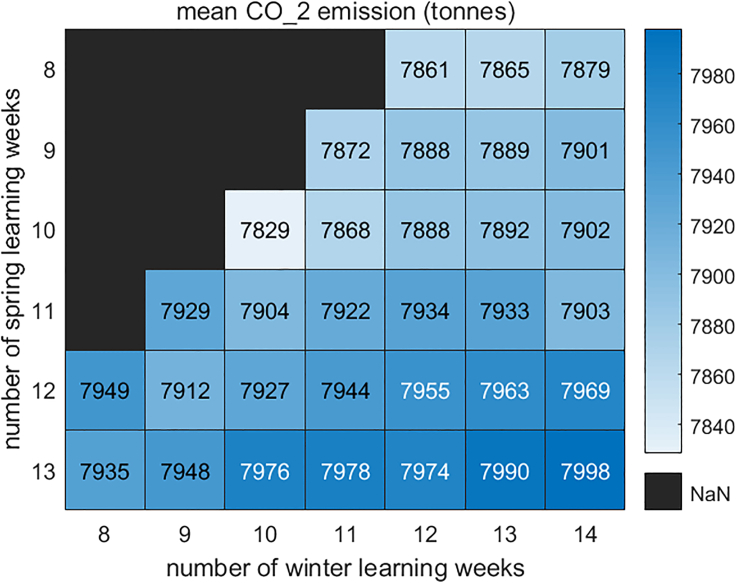
Winter classes and CO2 Emission (AEF)

**Figure 11:**
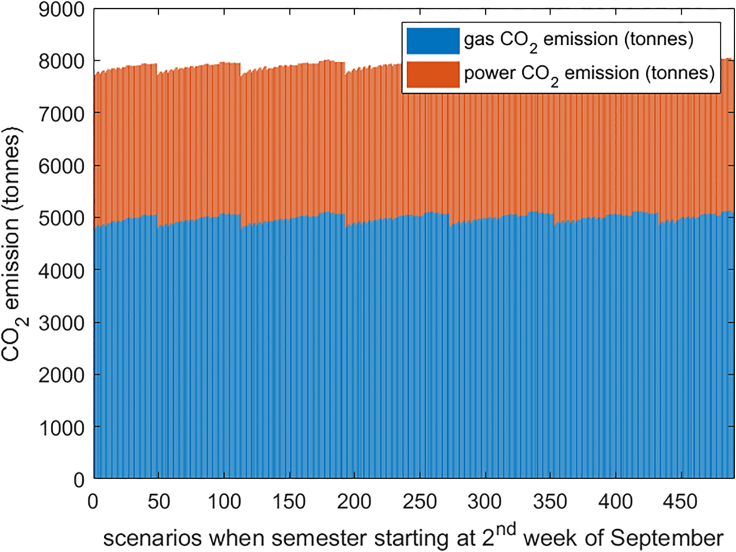
CO2 emission from gas and imported power (AEF)

If universities use a fixed emission factor to calculate the corresponding CO_2_ emissions, then the arrangement with minimum energy demand is naturally the one with the minimum CO_2_ emission. However, such a decision ignores the fact that the carbon factor is a spatiotemporal variable and therefore may greatly misestimate the actual CO_2_ emissions. For universities considering using AEF and MEF, minimum consumption does not exactly mean minimum CO_2_ emissions. Figure 9, for example, provides the relationship between CO_2_ emissions and loads for all semester cases starting in the second week in September. It can be seen that in the context of AEF, electricity demand and CO_2_ emission relationship become complicated although there are a number of distinct groupings. Because the heat demand is satisfied by natural gas, a fuel with a nominally fixed CO_2_ intensity, the linear relationship is basically unchanged. Figure 10 shows the emissions associated with various combinations of winter and spring learning weeks; as the amount of winter teaching rises, the average emissions climb regardless of the remaining schedule. Finally, irrespective of the schedule, two-thirds of the CO_2_ emissions are from natural gas (Figure 11).

It must be pointed out that the minimum CO_2_ emissions case in Figure 9, which is less than 7,700 metric tons per year, maybe an unattainable amount of carbon emissions in practice. This result corresponds to a specific arrangement (#113, Table S1) which splits the 30 learning weeks into equally long winter, spring, and summer sessions and would involve working over the New Year Eve.

#### Case 1: CO_2_ emissions with current UoE operational mode

Currently, UoE starts its semester in the third week of September, with a 14-week (sometimes 13 weeks) winter learning semester, followed by a 3-week vacation time (winter holiday and university closure period). Then, after a 12-week spring semester from the middle of January, and a 3-week spring holiday, the university enters into its exam month followed by the 15-week summer vacation. The total study weeks (including exams) are 30 weeks, and this arrangement will be referred to as the “Edinburgh Mode.”

**Figure 12:**
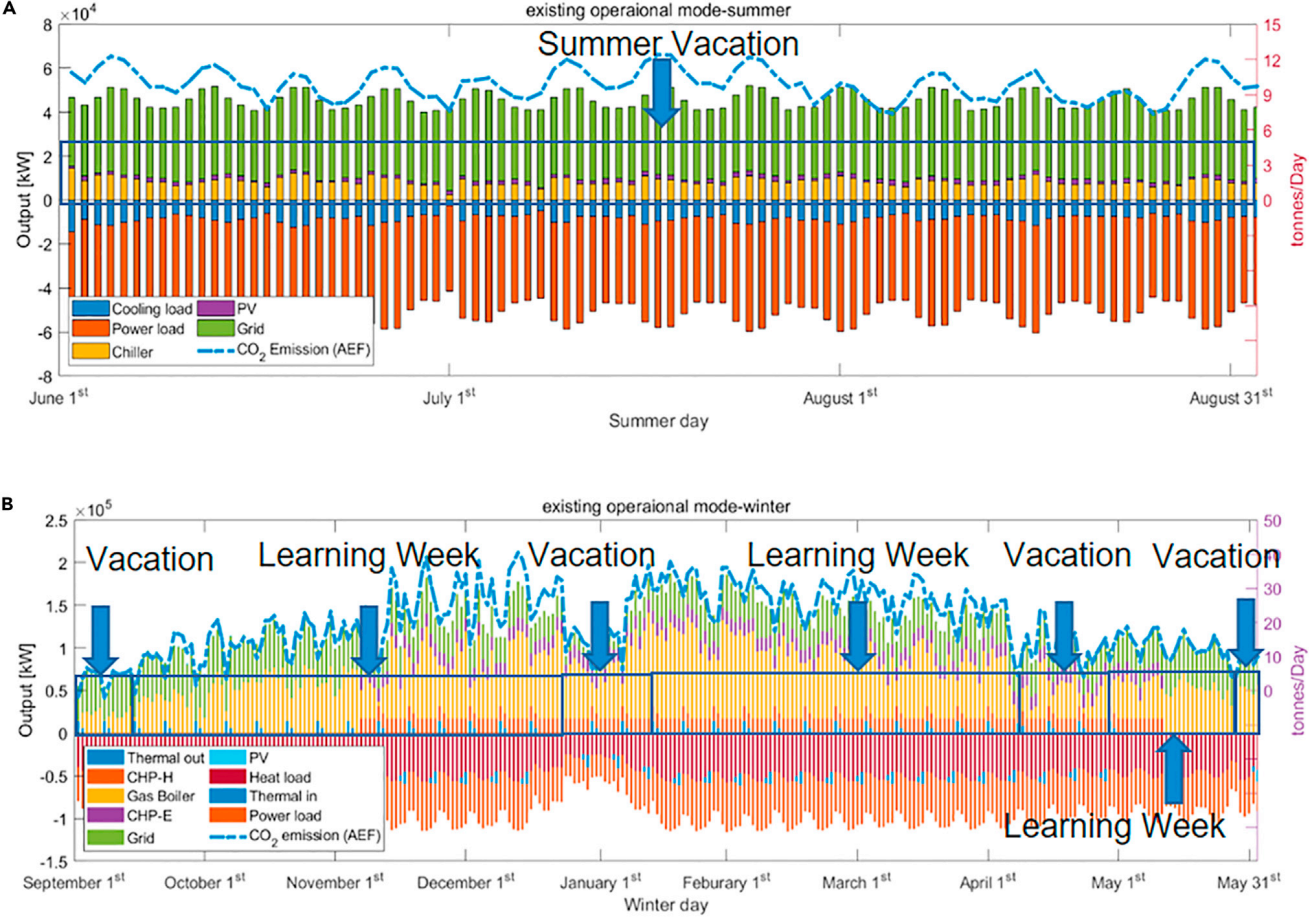
Existing operational mode details (A and B) (A) Summer details and (B) winter details.

Figure 12 illustrates the seasonal breakdown details of daily energy consumption in the current university operational mode. At present, the annual CO_2_ emission of the GS campus is 8,031 tons (AEF method).

#### Case 2: minimum CO_2_ emissions schedule with AEF

**Figure 13:**
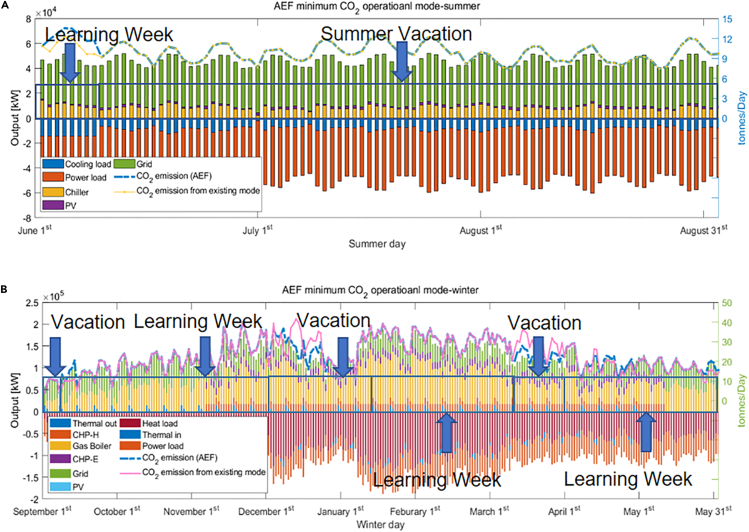
Minimum CO2 operational mode (A and B) (A) Summer and (B) winter (AEF).

Different semester schedules have a significant influence on annual energy consumption. When searching for the best acceptable operational mode, it is important to consider the “festival impact.” For example, Christmas and New Year Eve are important festivals on which there are no classes. The timing of Easter is less well defined but generally falls within the spring vacation. Therefore, any acceptable arrangement has to exclude these festivals as holidays. After comparing hundreds of scenarios, the best choice is to start the semester in the second week in September (Figure 13) with overall CO_2_ emissions of 7,864 tons/year, a 2% reduction from the existing situation, and around half of the maximum possible. From the results, it is clear that reducing activities in the winter would be essential to lower emissions; with December normally one of the coldest months in Edinburgh, a holiday arrangement is a wise choice, although this may well simply displace emissions to domestic dwellings.

Compared with the existing operational mode, the suggested arrangement, with minimum CO_2_ emissions, changes schedule as follows: a 12-week semester starts in the second week of September and a 6-week winter holiday (winter vacation plus university closure period), followed by an 8-week spring semester and a 3-week spring vacation. The rest of the year is divided into a 10-week summer semester and a 13-week summer vacation. In other words, compared with the existing “Edinburgh mode,” the proposed arrangement moves 4 learning weeks to summer and extends the winter vacation. This kind of arrangement is a combination of several existing university operational modes from Scotland to England.

#### Case 3: minimum CO_2_ emissions schedule with MEF

Due to various reasons outlined earlier, the MEF value may not be easily available to university operators; MEF is more complicated to calculate than AEF in a decentralized energy market, and local MEF value has not yet been published in the UK. But it is worth acknowledging that it better reflects the demand influence on CO_2_ emissions. As explained earlier, the national MEF value is used here but it would be expected that local MEF could be different from university to university.

**Figure 14:**
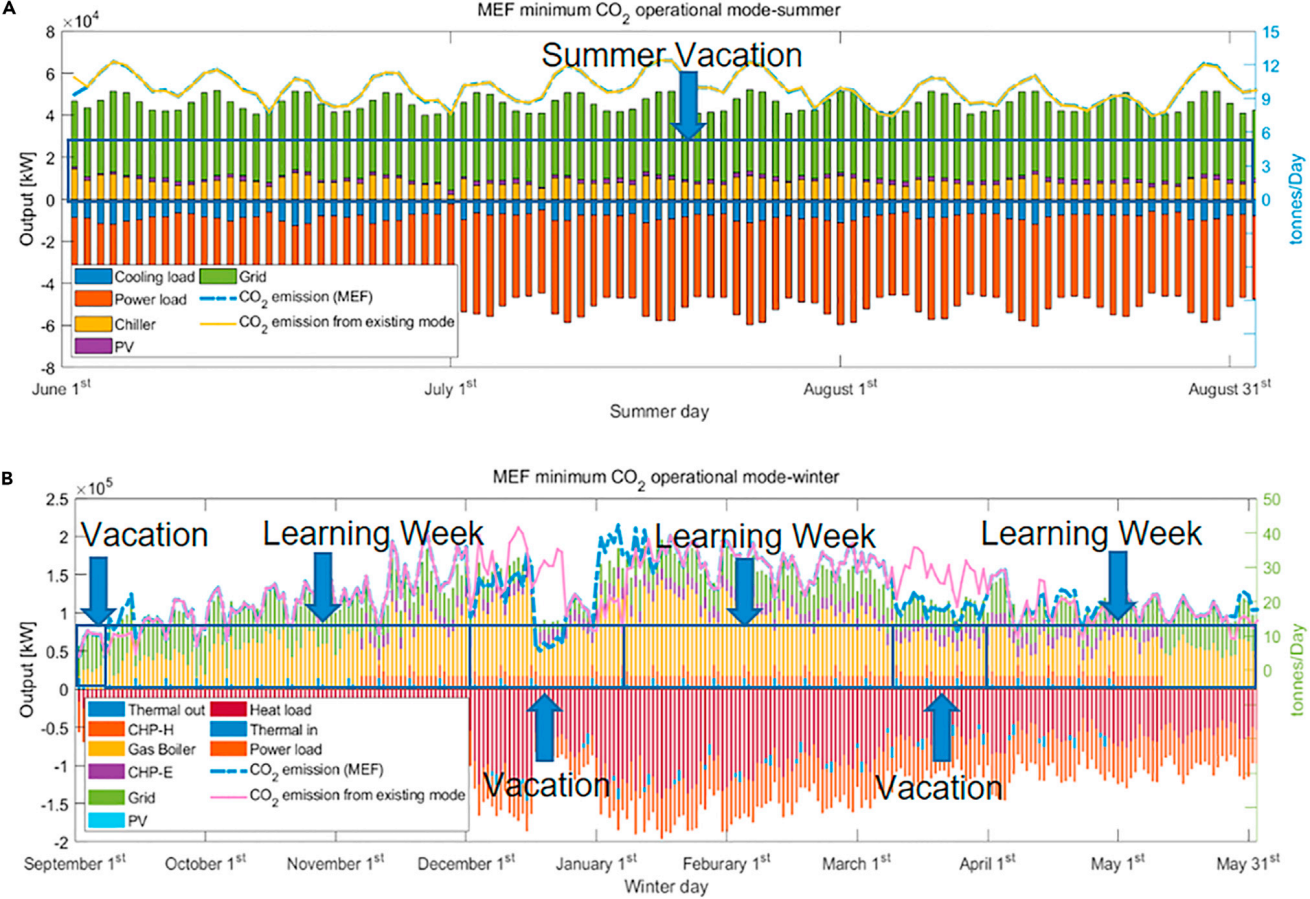
Minimum CO2 operational mode (A and B) (A) Summer and (B) winter (MEF).

There are significant differences in the semester arrangements suggested by using the MEF and AEF methods and their corresponding daily CO_2_ emissions distributions. From Figure 14, it is clear that compared with the AEF method the change in semesters has a greater impact on CO_2_ emissions under MEF. This is because when the electricity system operators balance supply and demand, coal and gas stations would often be switched on or off first, as these generators have a higher emission factor than the average grid level (Hawkes, 2010). Moreover, unlike the AEF method, MEF CO_2_ emission calculation is determined not only by the overall demand but also by the timing of that demand.

The lowest carbon pattern with the MEF method is close to the AEF result with slight modifications. It starts the new semester on the second week of September with a 12-week winter learning semester and a 5-week winter holiday (winter vacation, university closure). Then, the second semester begins in the second week of January and finishes at the start of March. The final three weeks in March are regarded as spring break. Finally, from the end of March is a 9-week semester with a summer vacation beginning in June. The annual CO_2_ emissions calculated by the MEF method is 7,698 tons, 167 tons smaller than with the AEF method, a reduction of 4.2%.

#### Main diversities among these scenarios

**Figure 15:**
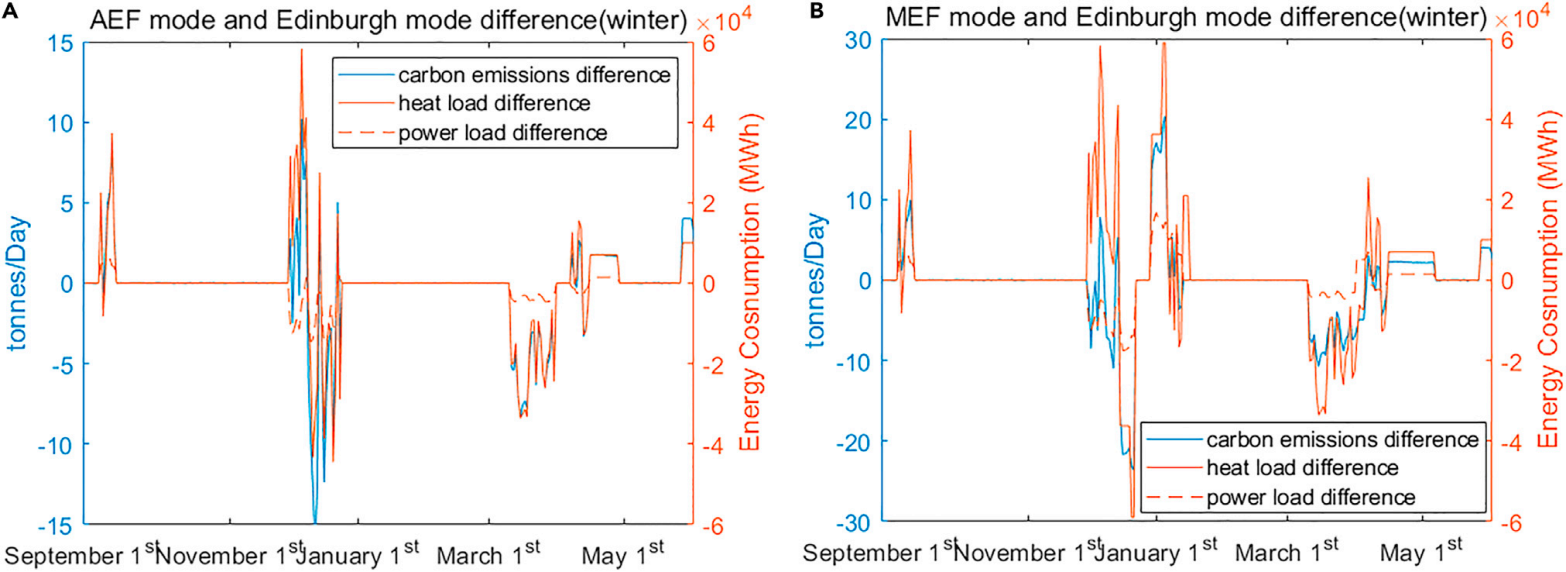
The carbon emission differences between different operational modes (A) AEF mode and Edinburgh mode main carbon emission difference. (B) MEF mode and Edinburgh mode main carbon emission difference.

Figure 15 illustrates the main differences between the three modes of operation. As can be seen from Figures 12A, 13A, and 14A, there is little change in summer between different modes of operation, so Figure 15 only shows the differences in winter. Specifically, Figures 15A and 15B highlight the main differences: (1) between AEF mode and Edinburgh Mode, and (2) MEF mode and Edinburgh Mode, respectively. As can be seen from Figure 15, the main reason for different carbon emissions among operating modes is the difference in load and, more specifically, the difference in heat load.

## Discussion

The analysis has usefully clarified a number of points. As can be observed from Figure 11, around one-third of CO_2_ emissions come from electricity imports and the rest are due to natural gas. Although the carbon emission from electricity import would get lower with the growth of power grid decarbonization, the existing CHP would have very limited contribution to further CO_2_ savings due to the use of natural gas: Figures 12, 13, and 14 illustrate that CHP does not operate when the grid-carbon intensity is lower than the gas-carbon intensity of 184 gCO_2_/kWh. However, although CHP produces a large amount of CO_2_, it can provide multiple loads, including heating, cooling, and electricity, and is currently indispensable.

University campus heat demands are mainly satisfied by the co-products of CHP, which increasingly will not be a wise choice with the increasing power grid decarbonization. It is clear that universities need to look at reliable clean sources of heating to reduce their CO_2_ emissions. This could be via hydrogen delivered by repurposing the gas system or perhaps in nearer term via ground or air sourced heat pumps powered by onsite generation or from the power grid. PV is less effective at high latitudes, and the 26 kW PV panels currently installed generate no more than 200 kWh of electricity per day even in the middle of summer. Compared with the huge demand of the GS campus, this will have only a marginal impact unless it is used along with heat pumps.

It seems that proper appreciation of dynamic power grid CO_2_ intensity offers additional value in reducing carbon emissions when combined with more flexible semester arrangements. This work suggests, university operators should use AEF when calculating their CO_2_ emissions but use MEF for understanding how the demand could be shifted, i.e., semester arrangement changed. Compared with existing operating modes, AEF and MEF approaches both recommended reduced activity in winter by starting the semester earlier and extending the winter vacation, due to the high value of AEF and MEF in that season. The results have clearly shown that MEF offers a marginally strong signal to decrease CO_2_ emissions but is more difficult to project.

Two different emission factors have been discussed in this work. They have their own merits and could be more suitable in different applications. Overall, for the national scale comparison study, we recommend that the universities use national AEF values for comparable results for two reasons. Firstly, the national MEF value is hard to obtain in practice because different locations may have different types of marginal generators. Secondly, considering the fact that the local marginal generator is dynamically determined by local power system real-time operations, it is hard to determine MEF value a year ahead for planning study. Thus, it is challenging for universities to obtain or estimate this value when discussing their semester arrangement impacts on carbon emissions. Compared with MEF, national AEF is easier to obtain (it can be easily calculated using public data from the UK national grid a year ahead when the university makes semester arrangement decisions) and is identical for all schools or other institutions in the country. Thus, the university efforts on reducing carbon emissions can be reviewed based on the same benchmark, i.e., the AEF value.

The university is a unique entity and its energy consumption is highly regulated by its semester schedule. Understanding the reasons behind energy consumption is crucial to reducing carbon dioxide emissions from campuses. This research can also help other universities and large organizations, across the UK and worldwide, think about how to reduce their carbon footprint by looking at their operational patterns. The methodology purposed in this paper can enhance university engagement in climate change, accelerating the carbon-neutral transformation of the universities in management, operations, and policy development. More importantly, the university's adoption of this method will bring great significance to society: changing semester arrangements to reduce carbon emissions can raise students' awareness of environmental protection and climate change, as it would be a direct and practical example for inspiring students to investigate the relationship between energy consumption pattern and carbon emissions. This could hopefully bring about a long-term impact on the construction of a low-carbon future.

### Conclusion and future work

This paper examines a conceptually simple approach to reduce campus CO_2_ emissions by changing semester structure. An energy hub model aimed at minimum CO_2_ emission for a real university campus is formulated. Along with the semester arrangement, AEF and MEF effects on CO_2_ calculations are being discussed, which do not appear in most existing studies. In the case study, the full potential university curriculum arrangements are compared to find the most environmentally friendly one for the UoE. Aimed at minimizing CO_2_ emissions, the model gives optimal power combinations in different operational modes.

As stated in the Discussion, it is clear that with the existing campus energy system, even shifting the semester arrangement to reduce the carbon emissions, there are still over 7,000 tons of emissions, with two-thirds of carbon emissions coming from gas-fired CHP. If the university is to substantially reduce energy consumption and emissions, it needs to change its sources of electricity and heat to low carbon sources further, i.e., renewable generation or hydrogen, to replace the CHP-based energy system.

This work would have wide implications for many universities around the world. Given the fact that traditional carbon reduction methods require significant investment and time to upgrade the energy system, changing the semester schedule could be a firmly attractive choice to reduce carbon emissions, because it requires less investment and is easier to implement. The general applicability of the proposed method, considering the energy systems' differences all over the world, needs a good understanding of the local power grid carbon intensity and its variations.

### Limitations of the study

As what could be seen in the methodology, the main limitation of the model, is the low data resolution. An energy hub model operated on daily basis may not be able to illustrate the finer details of campus-level energy consumption. Although, the model can correctly identify semester arrangement impact on the carbon emissions, a much precise model could be presented, if the updated hourly heat and electricity consumption from the university were available.

Another limitation is that the CO_2_ calculation model for the university ignores the shifted demand influence on the value of power grid AEF value. However, considering the fact that compared with the huge demand balanced by the power grid, the university's demand is tiny, such a simplification will have little impact on the final results.

In the future, with sufficient information on universities’ activity and energy consumption datasets, there are some unexplored and interesting questions worth discussing. For example, it is reasonable to assume that different types of universities (science and engineering, social science, comprehensive universities) may have different behavior patterns even under the same semester arrangements. It is interesting to see to what extent these differences would contribute to different carbon emissions. Secondly, given the fact that the growing number of universities are willing to play a critical role on the issue of climate change, it is useful to adapt our methodology to universities under different climate zone for comparison studies. The impact of climate on arrangement optimization is also a meaningful research topic. Last but not least, it would be valuable to analyze the hourly level space utilization for some universities with considerable spare teaching space if a more detailed dataset and information are available in the future.

## STAR★Methods

### Key resources table

**Table:** 

REAGENT or RESOURCE	SOURCE	IDENTIFIER
**Software**		

Matlab	(Matlab, 2018)	https://www.mathworks.com/
Yalmip	(Lofberg, 2004)	https://yalmip.github.io/
CPLEX	(IBM, 2015)	https://www.ibm.com/analytics/cplex-optimizer

**Other**		

All relevant data for modeling	This paper	Properly cited wherever applicable, available in main reference list of the manuscript
Energy facility technical information	Table 4	Calculated from real operational data from University of Edinburgh
carbon emissions factor (both AEF and MEF)	Staffell et al.	https://electricinsights.co.uk/
Solar radiation data	Pfenninger and Staffell (2016)	https://www.renewables.ninja/
Gas emission factor	Staffell et al.	https://electricinsights.co.uk/

### Resource availability

#### Lead contact

Further information and requests for data, resources and reagents should be directed to and will be fulfilled by the Lead Contact, Dr.Wei (w.sun@ed.ac.uk).

#### Materials availability

This study did not generate new unique physical materials.

### Method details

The details of LMSR (Linear model stepwise regression) model and the energy hub model are given here.

#### LMSR model

LMSR is a systematic method for adding and removing terms from a linear or generalized linear model based on their statistical significance in explaining the response variable. The method begins with an initial model, and then compares the explanatory power of incrementally larger and smaller models. LMSR has an internal process of choosing features, ‘the feature extraction process’. Based on a variables’ significance level obtained by different selection criteria, such as F-test, Akaike information criterion (AIC) and Bayesian information criterion (BIC), LMSR chooses a subset of *X* to establish the regression model.

There are three distinct approaches of establishing an LMSR model. Forward selection starts with no variables in the model and introduces the variable later by a chosen fit criterion. With backward elimination the initial model involves all candidate variables, and deletes the variable (if any) using a chosen model fit criterion. Bidirectional elimination is a combination of the first two methods that test which variables should be included or excluded. In this study, the F-test is used as variable selection criterion and bidirectional elimination is used with features that are selectively added to and removed from the regression model.

The first stage is to establish an initial regression model with a random single input feature $x_{r}$

$$y=w_{lmsr}^{r}x_{r}+b_{lmsr}+\varepsilon \;$$

where $x_{r}$ are the features being chosen by the regression model, $w_{lmsr}^{r}$ is the weight that is associated with individual features, $b_{lmsr}$ is the intercept, and ε is a vector of error terms.

The second stage is to identify a feature not currently in the model that ‘improves’ the regression. This requires each available term to be tested for significance: if the p-value of any terms is less than an entrance tolerance (*p-Enter*), the term with the smallest p-value is added. This is iterated several times until no additional feature meets the entrance criteria.

The next stage is to determine whether any of the existing terms in the model does not add value to the regression. Terms are again tested for significance for p-values that are greater than an exit tolerance (*p-Remove*, i.e., the hypothesis of a zero coefficient cannot be rejected). If this is the case, then the term with the largest p-value is removed and the assessment returns to the second stage. Otherwise, it stops.

At any stage, the function will not add a higher-order term if the model does not also include all lower-order terms that are subsets of the higher-order term. For example, the function will not try to add the term $x_{1}*x_{2}^{2}\;$unless both $x_{1}$ and $x_{2}^{2}\;$are already in the model. Similarly, the function will not remove lower-order terms that are subsets of higher-order terms that remain in the model. For example, the function will not try to remove $x_{1}$ or $x_{2}^{2}\;$ if $x_{1}*x_{2}^{2}\;$ remains in the model.

Depending on the terms included in the initial model, and the order in which the function adds and removes terms, the function might build different models from the same set of potential terms. However, a unique initial model or a different sequence of steps does not guarantee a better fit. In this sense, stepwise models are locally optimal, but might not be globally optimal. For the understanding and interpretation of the model, the LMSR model in this study is a "linear" model

LMSR model parameter settings (Mathworks, 2019)

**Table:** 

LMSR model	Model Type
'constant'	Model contains only a constant (intercept) term.
'linear'	Model contains an intercept and linear term for each predictor.
'interactions'	Model contains an intercept, linear term for each predictor, and all products of pairs of distinct predictors (no squared terms).
'purequadratic'	Model contains an intercept term and linear and squared terms for each predictor.
'quadratic'	Model contains an intercept term, linear and squared terms for each predictor, and all products of pairs of distinct predictors.

#### Energy hub model

##### The objective of the model

The model objective is to minimize the annual CO_2_ emission ($A_{CE}$) for a campus,

(Equation 1) $$MinA_{CE}=\sum_{t=1}^{T}\Delta t\sum_{g=1}^{G}c_{g}P_{g}^{t}$$

where $c_{g}$ is the equivalent emission coefficient of $g_{th}$type of energy consumption; Δt is one day with total T of 364 days (52 weeks); $P_{g}^{t}$ represents $g_{th}$ type of power output at time t.

##### Energy conversion model

A general energy conversion device is described by Equations 2 and 3 (Ma et al., 2018)

(Equation 2) $$P_{k,j}^{out,t}=\eta_{k}^{t}P_{k,i}^{in,t}$$

(Equation 3) $$P_{min,i}\leq P_{out,i}\leq P_{max.i}$$

where i,jare the indexes of energy types, i,j∈{ng,e,h,c}. ng,e,h,c are short for natural gas, electricity, heat load and cooling load individually. $P_{k,j}^{out,t}$ and $P_{k,i}^{in,t}$ denote the output power/input power of energy converter k at time slot t. $\eta_{k}^{t}$ denotes the energy conversion efficiency of converter k at time slot *t.* More specifically, there are three energy conversion technologies being used in this paper, GB, CHP, and AC.

###### Gas boiler model

The GB heat output $P_{gb,h}^{out,t}$ equals the natural gas consumed by gas boiler $P_{gas,g}^{in,t}$ multiplies its efficiency $\eta_{gb}^{t}$:

(Equation 4) $$P_{gb,h}^{out,t}=\eta_{gb,h}^{t}P_{gb,g}^{in,t}$$

Also, the heat output $P_{gb,h}^{out,t}$ should within its operation range [$P_{gb,h}^{outmin,t}$,$P_{gb,h}^{outmax,t}$],

(Equation 5) $$P_{gb,h}^{outmin,t}\leq P_{gb,h}^{out,t}\leq P_{gb,h}^{outmax,t}$$

##### Absorption chiller

Similarly, the AC output $P_{ac,c}^{out,t}$ equals the heat fed into the AC from CHP,$P_{chp,h}^{out,t}$, multiplies its efficiency$,\eta_{ac,c}^{t}$

(Equation 6) $$P_{ac,c}^{out,t}=\eta_{ac,c}^{t}P_{chp,h}^{out,t}$$

The output should within its operation range as well:

(Equation 7) $$P_{ac,c}^{outmin,t}\leq P_{ac,c}^{out,t}\leq P_{ac,c}^{outmax,t}$$

##### CHP model

CHP electricity output $P_{chp,e}^{out,t}$ and heat output $P_{chp,h}^{out,t}$ equals the from gas turbine, multiplies its electrical (heat) efficiency, $\eta_{chp,e}^{t}$ and $\eta_{chp,h}^{t}$

(Equation 8) $$P_{chp,e}^{out,t}=\eta_{chp,e}^{t}P_{chp,g}^{in,t}$$

(Equation 9) $$P_{chp,h}^{out,t}=\eta_{chp,h}^{t}P_{chp,g}^{in,t}$$

The electricity and heat output should within its operation range [$P_{chp,e}^{outmin,t}$, $P_{chp,e}^{outmax,t}]$ and [$P_{chp,h}^{outmin,t}$, $P_{chp,h}^{outmax,t}$]

(Equation 10) $$P_{chp,e}^{out\min,t}\leq P_{chp,e}^{out,t}\leq P_{chp,e}^{out\max,t}$$

(Equation 11) $$P_{chp,h}^{out\min,t}\leq P_{chp,h}^{out,t}\leq P_{chp,h}^{out\max,t}$$

##### PV output model

The output power of PV panels can be expressed as follows:

(Equation 12) $$P_{k,e}^{out,t}=\eta_{overall}\cdot N_{k}\cdot P_{STC}\cdot G_{t}/G_{STC}$$

where $\eta_{overall}$ is the overall conversion efficiency of the system, $N_{k}$ is the area of PV panels, $G_{t}\;$is the solar radiation intensity at time t (W/m^2^), and $P_{STC}$ is the panel output at standard test conditions (irradiation, $G_{STC}$, of 1000 W/m^2^ and a cell temperature of 25°C). However, the actual operating conditions will always be different from it, thus the overall performance will be influenced (King et al., 2004). For practical reasons, the various factors, such as non-STC corrections, transposition factors, transformer losses, losses due to soiling of the panels, cable loss etc., that influence the performance, are often complied into a single factor, called the performance ratio, $R_{f}^{p}$. The overall efficiency can be represented as Equation 13. $\eta_{in}$ is the conversion efficiency of the PV panels.

(Equation 13) $$\eta_{overall}=\eta_{in}\cdot R_{f}^{p}$$

##### Energy storage model

An electrical or thermal energy storage device can be regarded as a load/energy source when it charges/discharges (Ma et al., 2018).

(Equation 14) $$E_{k,i}^{t+1}=\left(1-\delta_{k,i}\right)E_{k,i}^{t}+\left(\eta_{k,i}^{c}P_{k,i}^{in,t}-\frac{P_{k,j}^{out,t}}{\eta_{k,i}^{d}}\right)\Delta t$$

(Equation 15) $$0⩽P_{k,i,c}^{t}⩽u_{k,i}\cdot P_{k,i,c}^{max}$$

(Equation 16) $$0⩽P_{k,i,d}^{t}⩽\left(1-u_{k,i}\right)\cdot P_{k,i,d}^{max}$$

(Equation 17) $$E_{k,i}^{min}⩽E_{k,i}^{t}⩽E_{k,i}^{max}$$

(Equation 18) $$E_{k,i}^{week\;end}=E_{k,i}^{week\;start}$$

where i has the same meaning as in Equation 3, which indicates types of energy storages. $\delta_{k,i}\;$is the standby energy loss ratio of $k_{th}$ energy storage device in typei, whose charging and discharging efficiency are $\eta_{k,i}^{c}$ and $\eta_{k,i}^{d}$, respectively. In addition, $P_{k,\;i,c}^{t}$ and $P_{k,i,d}^{t}\;$are charging and discharging power of energy storage device k (type i) at time slot *t*, separately. $E_{k,i}^{t+1}$ and $E_{k,i}^{t}$ denote the energy stored at energy storage device k at time slots t+1 and t, individually.

Equation 14 presents the state of charge in the storage during time interval Δt before and after charging/discharging. $P_{k,i,c}^{max}$ and $P_{k,i,d}^{max}\;$denote the maximal charging/discharging power rate of energy storage device *k*, respectively, which cannot be exceeded when charging/discharging (Equations 15 and 16). Moreover, $u_{k,i}$ is binary (0–1) variable to guarantee the charging and discharging process will not happen simultaneously. Equation 17 is the upper and lower limit on storage. Moreover, for the sake of regulating that no energy is accumulated over time and ensuring the continuity of scheduling, Equation 18 ensures the stored energy toward the finish of the dispatch period should be equivalent to its initial value.

##### Energy balance constraints

The left side of Equations 19, 20, and 21 are the aggregate of output powers of all the power generation devices and the right side denotes the sum of all the power loads at time slot *t*. Clearly, the first equation represents the electric power balance, while the second and third refer to the heating and cooling power balance, respectively.

(Equation 19) $$\sum_{k=1}^{K_{e}}P_{k,e}^{out,t}=\sum_{l=1}^{L_{e}}P_{e}^{l,t}$$

(Equation 20) $$\sum_{k=1}^{K_{h}}P_{k,h}^{out,t}=\sum_{l=1}^{L_{h}}P_{h}^{l,t}$$

(Equation 21) $$\sum_{k=1}^{K_{c}}P_{k,c}^{out,t}=\sum_{l=1}^{L_{c}}P_{c}^{l,t}$$

##### AEF and MEF calculation

The background briefly presents the definitions of AEF and MEF respectively, here, Equations 22 and 23 demonstrate mathematical formulas to calculate them. AEF is calculated from:

(Equation 22) $$AEF_{t}=\left(\sum_{j}^{J}\left(G_{tj}*C_{j}^{e}\right)\right)/D_{t}$$

where $AEF_{t}$ refers to the AEF value in a given time period of *t.* $G_{tj}\;$represents the amount of generation from generation technology *j*, in the time period *t;* $C_{j}^{e}$ means the corresponding carbon emission factor of the generation technology *j*.$\;D_{t}$ is the demand, met by these generation technologies, at time *t.*

Similarly, MEF is calculated from:

(Equation 23) $$MEF_{t}=\left(\sum_{i}^{I}\left(G_{ti}^{m}*C_{i}^{e}\right)\right)/D_{t}^{s}$$

where $MEF_{t}$ refers to the MEF value in a given time period of *t.* Similarly to AEF, the $G_{ti}^{m}\;$represents the amount of generation from the marginal generation technology *i*,(defined as those which are flexible and dispatchable) that react to the shifted demands$\;D_{t}^{s}$; and $C_{i}^{e}$ is the corresponding emission factor of that generation technology *i*.

Equation 22 is useful for universities which would like to use AEF to calculate their CO_2_ emissions. $E_{CO_{2}}^{AEF}$ refers to the total CO_2_ emission in the AEF method, which equals the sum of emissions from different energy types:

(Equation 24) $$E_{CO_{2}}^{AEF}=\sum_{t}^{T}\left(AEF_{t}\cdot P_{t}^{e}+EF_{t}^{ng}\text{∗}P_{t}^{ng}\right)$$

where power from the power grid $P_{t}^{e}$ and the imported natural gas $P_{t}^{ng}$, multiplied by its corresponding emissions factor,$\;AEF_{t}$, and emission factor of natural gas $EF_{t}^{ng}$.

Unlike AEF, MEF is appliable only in the scenario with demand shifting. When MEF is implemented to calculate the CO_2_ emission (Equation 23), the current power generation combination of existing operational mode, $m_{e},\;$ i.e., the mode before demand shifting, must be calculated first. Then, the shifted electricity demand $D_{t}^{e_{s}}$and the shifted natural gas demand, $D_{t}^{ng_{s}},\;$multiply the corresponding MEF value, $MEF_{t}$ and $EF_{t}^{ng}$, to obtain a CO_2_ emissions difference between two operational modes, $m_{e}$and $m_{s}$. The MEF calculated carbon emissions,$\;E_{CO_{2}}^{MEF}$, is given by the AEF calculated CO_2_ emission plus this difference $E_{CO_{2}}^{MEF}$.

(Equation 25) $$E_{CO_{2}}^{MEF}=E_{CO_{2}}^{AEF}+\sum_{t}^{T}\left(MEF_{t}\cdot D_{t}^{e}+EF_{t}^{ng}\text{∗}D_{t}^{ng}\right)$$

(Equation 26) $$D_{t}^{e}=P_{t\left(m_{e}\right)}^{e}-P_{t\left(m_{s}\right)}^{e}$$

(Equation 27) $$D_{t}^{ng}=P_{t\left(m_{e}\right)}^{ng}-P_{t\left(s\right)}^{ng}$$

where $P_{t\left(me\right)}^{e}$ $\left(P_{t\left(me\right)}^{ng}\right)$ and $P_{t\left(ms\right)}^{e}$ $\left(P_{t\left(ms\right)}^{ng}\right)$ refer to import power (natural gas) from power grid (gas network) at time *t* in two operational modes, $m_{e}$ and $m_{s}$.

Let's consider a simple case: assuming in one day, from 8:00 to 8:30 am, there are wind farm (assuming 0 gCO2/kWh (Hawkes, 2010)), Gas (assuming 600 gCO2/kWh (Hawkes, 2010)) and Coal power station (assuming 900 gCO2/kWh (Hawkes, 2010)) in the market to balance the total electricity demand, with a market share of 30%,40% and 30% respectively, then the AEF is:

600 ∗ 40% + 900 ∗ 30% + 0 ∗ 30% = 510 gCO_2_/kWh

If the end-user decides to increase its demand by 1MWh and the coal power station responds to that, then the MEF value equals the emission factor from the station, which is 900 gCO2/kWh.

Although the methodology seems straightforward, in reality, it could be challenging to finish the calculation in large systems. Such calculations for the UK power network require the efficiency of each generator type to determine fuel consumptions and corresponding carbon amount, but the efficiency of each generator is not a fixed figure. For instance, the part-load efficiency of thermal power plants is often lower than its rating value (Roeder and Kather, 2014). Secondly, in the UK, there is imported electricity from other countries, e.g., France and Ireland, whose emission factors are difficult to calculate at high temporal resolution, i.e., half-hourly level or daily level (Cleary et al., 2016). In addition, different potential methodologies will determine different historical MEF as the definitions of which plant is marginal in any given settlement period are different (Lane Clark & Peacock LLP, 2015). Also, calculating MEF or AEF requires a model to describe the Balancing Mechanism of the Great Britain electricity market, which is well outside the scope of this study. Therefore, this work does not engage with providing the entire AEF and MEF model. Instead, it uses data from a developed peer-reviewed model, the Drax Electric Insights, which was established by the Drax group (Staffell et al., 2017).

##### Semester arrangement constraints

The semester arrangement needs to follow some constraints, which are used to generate a reasonable result. These constraints are formed according to the historical records of semester dates from UK universities, to ensure they are subject to existing common arrangement.

(Equation 28) $$S_{t}^{d}=Monday$$

(Equation 29) $$Sept.08\;⩽S_{t}^{d}⩽Oct.08$$

(Equation 30) $$L_{w}^{n,min}⩽L_{w}^{n}⩽L_{w}^{n,max}$$

(Equation 31) $$L_{w}^{N,MIN}⩽\;\sum_{\text{n}}^{\text{N}}L_{w}^{n}\;⩽L_{w}^{N,max\;}$$

(Equation 32) $$H_{w}^{n,min}⩽H_{w}^{n}⩽H_{w}^{n,max}$$

(Equation 33) $$\sum_{\text{n}}^{\text{N}}L_{w}^{n}+\sum_{\text{n}}^{\text{N}}L_{w}^{n}={\text{N}}_{\text{week}}^{\text{y}}$$

Equations 29 and 30 indicate the restriction on the start date of the school year: the start date of the school year should be Monday and range from September 8^th^ to October 8^th^ each year. Equations 30 and 31 are used to regulate the length and total number of learning weeks, where $L_{w}^{n}$ refers to every kind of learning week, $L_{w}^{n}\in$ (spring learning week; winter learning week and summer learning week), that need to be greater than the minimum number of weeks $L_{w}^{n,min}$, and also less than the maximum number of weeks $L_{w}^{n,max}$. In addition, the total number of study weeks should be within a reasonable range [$L_{w}^{N,MIN}$,$L_{w}^{n,max}$].

Similar, Equation 32 refers to the duration of each holiday, $H_{w}^{n}$, $H_{w}^{n}\in$ (winter vacation, university closure, spring vacation and summer vacation), should be within a range of $H_{w}^{n,min}$ to $H_{w}^{n,max}$. Finally, the sum of learning weeks $\sum_{\text{n}}^{\text{N}}L_{w}^{n}$and the sum of holidays $\sum_{\text{n}}^{\text{N}}L_{w}^{n}$ should equal to the number of weeks in a year, ${\text{N}}_{\text{week}}^{\text{y}},$which is 52 in this paper. Moreover, in our model, we also consider the impact of traditional holidays. After generating these arrangements automatically, those do not meet the conditions below (Equations 34 and 35) are manually eliminated to ensure that the conclusion of the model is reasonable. The conditions we examine are:

(Equation 34) Christmas∈(WinterVacation;UniversityClosure)

(Equation 35) Atleastoneweekofspringvacation∈April

These two conditions are used to regulate that 1). Christmas should locate in winter vacation or university closure period. 2) Considering it is difficult to estimate the date of Easter day, condition 35 assumes the Easter day and the holiday, is in April.

## Data Availability

The energy consumption data is business confidential data of the university of Edinburgh. The rest of the input data are available in the key resources table and in the body of the text. The M-script files are available for academic purposes upon reasonable request. Any additional information required to reanalyse the data reported in this paper is available from the lead contact upon request.

## Supplemental information

Table S1. List of potential semester arrangements, related to Table 5
